# Complete Genome Sequence of Bacteriophage EC6, Capable of Lysing *Escherichia coli* O157:H7

**DOI:** 10.1128/genomeA.00085-12

**Published:** 2013-01-31

**Authors:** Birendra R. Tiwari, Jungmin Kim

**Affiliations:** Department of Microbiology, School of Medicine, Kyungpook National University, Daegu, Republic of Korea

## Abstract

A bacteriophage, EC6, capable of lysing *Escherichia coli* serotype O157:H7 was isolated and found to be a member of the family *Myoviridae*. The genomic sequence of phage EC6 was composed of 86,231 bp with a G+C content of 38.9% and is very similar to the sequences of *Escherichia* phage wV8 and *Salmonella* phage Felix O1.

## GENOME ANNOUNCEMENT

*Escherichia coli* serotype O157:H7 is a major pathogen that causes diarrhea, colitis, and hemolytic-uremic syndrome (1, 2). We isolated a novel *E. coli* O157:H7 bacteriophage (phage), EC6, from a sewage sample. Several clinical isolates of *E. coli* were tested to determine the phage’s host spectrum. Phage EC6 might form lytic plaques against sorbitol-nonfermenting *E. coli* but not against sorbitol-fermenting strains. Transmission electron microscopic images revealed that the EC6 phage belongs to the family *Myoviridae*. The sequencing of the phage EC6 genome was performed by combining the 454 and Illumina sequencing platforms using Genome Sequencer (GS) assembler 2.6 (Roche Diagnostics, Branford, CT) and CLC Genomics workbench 5.5 (CLCbio, Denmark), respectively. The resulting contigs were assembled through manual curation using CodonCode aligner v3.7.1 (CodonCode Corp., Dedham, MA). The final contigs were remapped with raw reads to correct errors, and dubious regions were masked as gaps. Genes, tRNA, and rRNA were identified using Glimmer, tRNAscan-SE, and HMMER programs along with EzTaxon-e rRNA profiles (3–6). The identified genes were annotated with RefSeq, catFam, Clusters of Orthologous Groups (COG), SEED, and GenBank databases (7–11). The genome of phage EC6 was composed of 86,231 bp with a G+C content of 38.9% and 136 open reading frames (ORFs) that encoded 16.9% functionally known proteins and 83.1% hypothetical proteins. In addition to genes for structural and general functions, the genome was composed of genes encoding a full set of enzymes related to replication, recombination, modification, transport, and metabolism. Based on COG database categories, proteins for the following functions can be predicted: structural and general functions (phage capsid and scaffolds, terminase, Mu-like prophage protein, putative phosphatase, muraminidase), replication, recombination, and repair (ATP-dependent DNA ligase, DNA polymerase I, replicative DNA helicase), transcription regulation (predictive transcriptional regulator), posttranslational modification (periplasmic serine protease homologous to putative head maturation protease of phage wV8, glutaredoxin, organic radical activating enzyme, molecular chaperone-HSP90 family), nucleotide transport and metabolism (thymidylate synthase, ribonucleotide reductase alpha-subunit, ribonucleotide reductase beta-subunit, oxygen-sensitive ribonucleoside-triphosphate reductase, phosphoribosyl pyrophosphate synthetase), coenzyme transport and metabolism (dihydrofolate reductase, nicotinic acid phosphoribosyl transferase), and an unknown function (putative phage Mu protein). In addition, the hypothetical protein AFU62408, which is adjacent to DNA polymerase, is 99% similar to the DNA polymerase of *Salmonella* phage SPT-1, which is likely the large subunit of DNA polymerase. Based on the functions of the encoded proteins, the phage EC6 genome is closely similar to that of *Escherichia* phage wV8 (GenBank accession no. NC_012749) (6) and that of *Salmonella* phage Felix O1 (GenBank accession no. NC_005282.1) (7). The phage EC6 genome differed from the genomes of both of these phages in its lack of genes encoding tail fiber proteins. However, the phage EC6 capsid and scaffold proteins are 97% similar to the tail fiber protein GP37 of phage wV8, with minor differences at the C-terminal region.

In conclusion, *E. coli* O157:H7-specific phage EC6 is a member of the *Myoviridae* family and is closely related to the *Escherichia* phage wV8 and *Salmonella* phage Felix O1.

### Nucleotide sequence accession number.

The complete genome sequence of phage EC6 has been stored in GenBank under the accession no. JX560968.
